# Folding, Assembly, and Persistence: The Essential Nature and Origins of Biopolymers

**DOI:** 10.1007/s00239-018-9876-2

**Published:** 2018-11-19

**Authors:** Calvin M. Runnels, Kathryn A. Lanier, Justin Krish Williams, Jessica C. Bowman, Anton S. Petrov, Nicholas V. Hud, Loren Dean Williams

**Affiliations:** School of Chemistry and Biochemistry, Georgia Institute of Technology, Atlanta, GA 30332 USA

**Keywords:** Polynucleotide, Protein, Carbohydrate, Folding, Assembly, Self-complementarity

## Abstract

Life as we know it requires three basic types of polymers: polypeptide, polynucleotide, and polysaccharide. Here we evaluate both universal and idiosyncratic characteristics of these biopolymers. We incorporate this information into a model that explains much about their origins, selection, and early evolution. We observe that all three biopolymer types are pre-organized, conditionally self-complementary, chemically unstable in aqueous media yet persistent because of kinetic trapping, with chiral monomers and directional chains. All three biopolymers are synthesized by dehydration reactions that are catalyzed by molecular motors driven by hydrolysis of phosphorylated nucleosides. All three biopolymers can access specific states that protect against hydrolysis. These protected states are *folded*, using self-complementary interactions among recurrent folding elements within a given biopolymer, or *assembled*, in associations between the same or different biopolymer types. Self-association in a hydrolytic environment achieves self-preservation. Heterogeneous association achieves partner-preservation. These universal properties support a model in which life’s polymers emerged simultaneously and co-evolved in a common hydrolytic milieu where molecular persistence depended on folding and assembly. We believe that an understanding of the structure, function, and origins of any given type of biopolymer requires the context of other biopolymers.

Polymers are large molecules formed by covalently linking small monomers into chains. Polyethylene, for example, is a synthetic polymer with molecular formula (–C_2_H_4_–)_n_ and molecular weight around 5 million Daltons that is used to make plastic bottles and bags. Living systems are united by their expression and utilization of three types of polymers. These three biopolymers, the subject of this paper, are polynucleotide (DNA and RNA), polypeptide (protein), and polysaccharide (polymerized sugars). Biopolymers have special properties that distinguish them from other polymers.

Biopolymers:

(i) spontaneously fold and assemble into precise and highly elaborate yet fragile assemblies with meager stabilities,
(ii) spontaneously degrade by hydrolysis in the aqueous environments characteristic of biological systems,
(iii) are self-protective against hydrolysis (by folding) and partner-protective (by heterogeneous assembly).

The three biopolymer types differ profoundly in their properties and functions. Polypeptide and polynucleotide dominate the functional and informational machineries of life, while polysaccharide is important in physical structure, energy storage, and recognition. The three biopolymers occupy discrete chemical spaces. Yet, biopolymers share many critical “universalities.” An understanding of structure, function, and origins of a given biopolymer type requires recognition of these universalities and the context of the other two biopolymer types.

Universalities of biopolymers include the ability to fold and assemble spontaneously. All three biopolymer types are self-complementary and pre-organized. Biopolymer self-complementarity is conditional and can be switched on and off by sequence, composition or linkage chemistry. Biopolymers are chemically unstable in aqueous media but persist for long periods via kinetic trapping. The depths of these kinetic traps are modulated by folding and assembly. Biopolymers are chiral and directional, and are synthesized by condensation dehydration using phosphorylated intermediates in reactions mediated by divalent cations and driven by phosphate dependent motors. Lipids are not discussed here because they are not covalent polymers. However, our conclusions would be unaltered by their inclusion.

## Chemical Cousins

In contrast to the usual approach of analyzing each biopolymer separately, we focus first on phenomena that are common to all biopolymers (Table 1). We use the same nomenclature to describe a given phenomenon or characteristic without regard to the type of biopolymer.

**Table 1:** Biopolymer Universalities and Idiosyncrasies

Attribute	Polynucleotide	Polypeptide	Polyglucose
Primary proficiency	Maintain, record, and transduce information, catalyze chemical reactions	Catalyze and regulate chemical reactions, provide physical structure	Provide physical structure, energy storage, and recognition
Conditional self-complementarity^U^	Yes	Yes	Yes
Condition for self-complementarity	Nucleotide sequence	Amino acid composition	Linkage stereochemistry (β- vs. α-anomer)
Small number of types of folding elements^U^	Yes	Yes	Yes
Folding element identities	Nitrogenous bases	Peptide linkage	Cyclic glucose
Enzymatic capability	Moderate	High	Low
Sidechain diversity	Low: four planer nitrogenous bases	High: 20 amino acid sidechains	N/A: no sidechains
Sidechain complementarity	Yes, base pairing	No	No
Backbone self-complementarity	No: anionic, self-repulsive backbone	Yes: neutral, cohesive backbone	Yes: neutral, cohesive backbone
Complementary hydrogen bonding^U^	Unipolar, coplanar	Unipolar, coplanar	Bipolar, non-planar
Net hydrogen bond polarity	Large excess of acceptors over donors	Equivalent number of acceptors and donors	Excess of acceptors over donors
Selective self-interaction of hydrolyzed monomers^U^	No	No	No
Backbone linearity	Yes	Yes	Sometimes
Strand directionality^U^	Yes, 5′–3′	Yes, N to C	Yes, 1–4
Secondary structure^U^	Helices, bulges, stem-loops, pseudoknots, etc	α-Helices, β-sheets and turns	Elongated fibers
Conformational constraints^U^	“Rigid nucleotides,” planar bases	Planar peptide, allowed regions of ϕφ space	Conformational preferences within and between cyclic glucose
Self-destruct mechanism	RNA: Yes (2′ hydroxyl) DNA: No	No	No
Required folding cofactors	Cations	None	None
Degradation by hydrolysis^U^	Yes	Yes	Yes
Polymerized by	Protein	Ribozyme RNP complex	Protein
Polymerization is dependent on divalent cations^U^	Yes	Yes	Yes
Polymerization mechanism^U^	Condensation dehydration	Condensation dehydration	Condensation dehydration
Polymerization intermediates^U^	Phosphorylated	Phosphorylated	Phosphorylated
Driver of polymerization motor^U^	Phosphate release	Phosphate release	Phosphate release
Retention of phosphate during polymerization	Yes	No	No

*U* indicates universal property of all biopolymers

### Biosynthesis

**Fig. 1:**
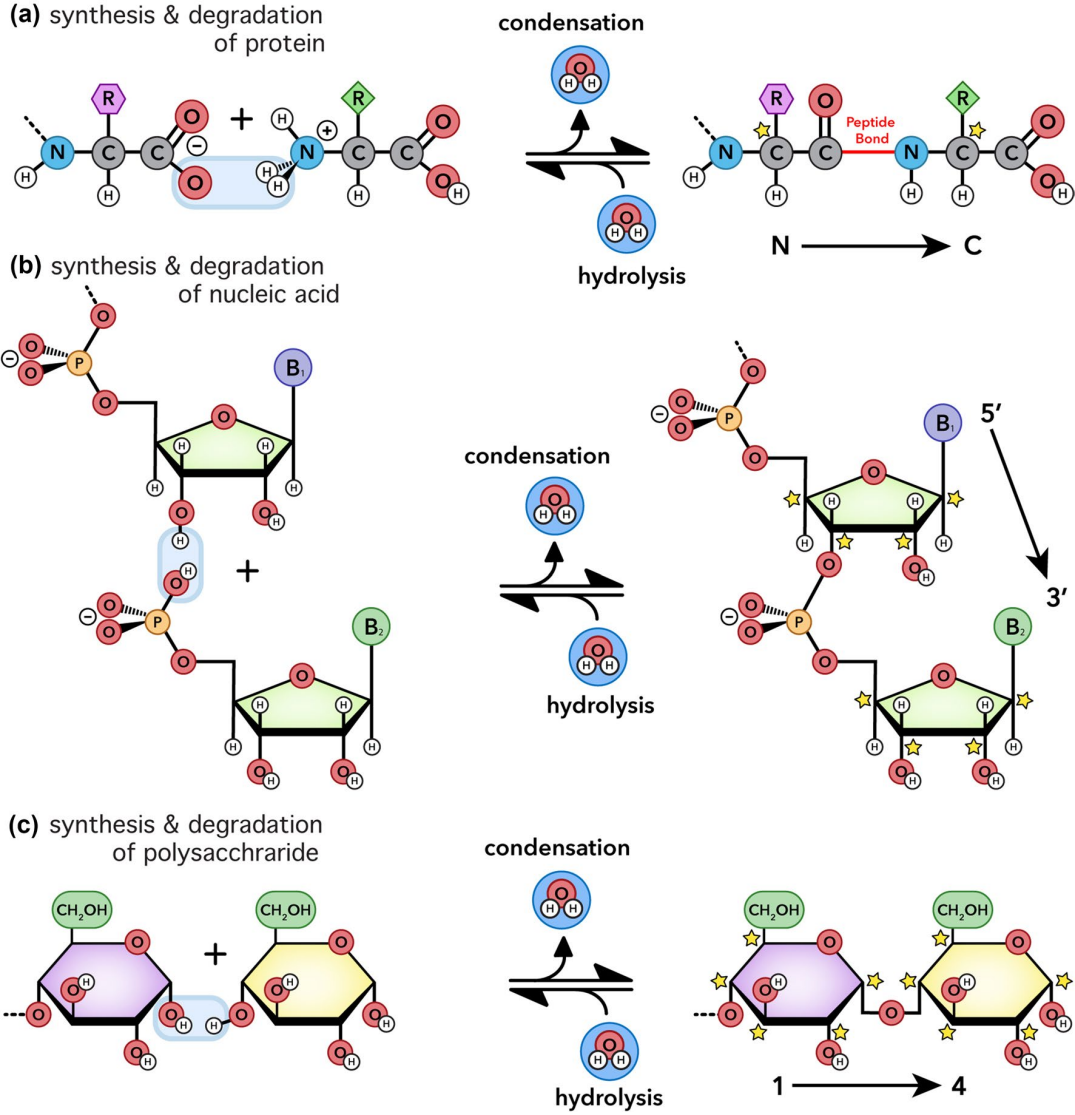
Net reactions for biopolymer formation by condensation dehydration and biopolymer degradation by hydrolysis. **a** Protein. **b** RNA. **c** Polysaccharide. All biopolymers are chiral and directional with distinctive ends. Chiral centers (stars, shown in polymers only) and strand directionalities (arrows) are indicated. Blue boxes indicate (in toto) the atoms involved in the synthesis/degradation reactions. (Color figure online)

Biopolymers are universally formed by condensation dehydration reactions, which release water (Fig. 1) to link well-defined and modest sets of monomers. Proteins are formed by condensation of 20 types of amino acids. Polynucleotides are formed by condensation of four types of nucleotides. Cellulose, the most abundant polymer in the biosphere, is formed by condensation of one type of monomer—glucose (McNamara et al. 2015). Complex cell-surface polysaccharides contain fewer than 20 different monosaccharides (Gabius et al. 2011). Here we will limit our discussion of polysaccharide to polyglucose, encompassing cellulose, glycogen, amylose, amylopectin, and chitin (acetylated glucose). However, our conclusions apply to polysaccharide in general.

**Fig. 2:**
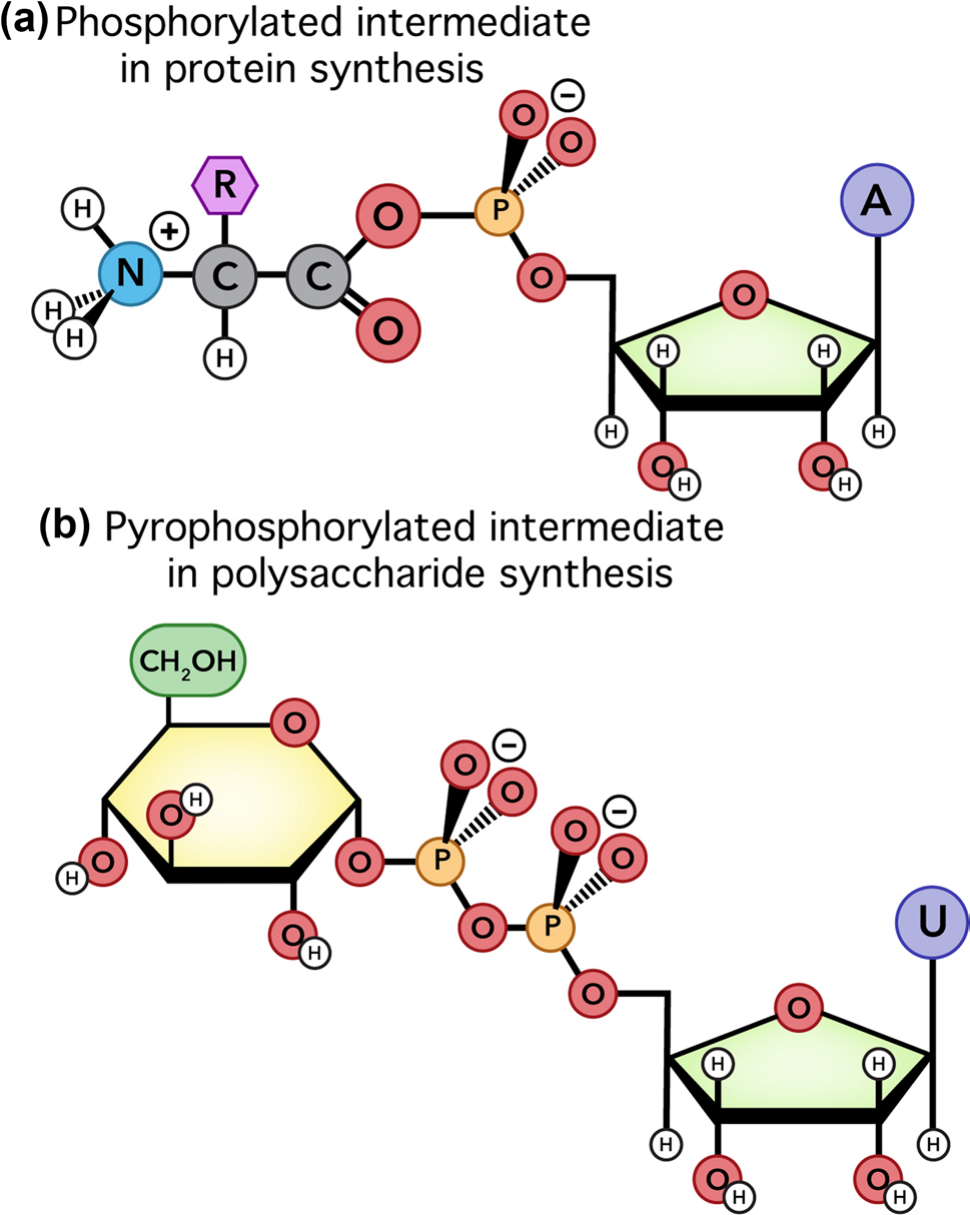
Intermediates in the biosynthesis of **a** protein and **b** polyglucose

Another universal property of biopolymers is their synthesis via phosphorylated or pyrophosphorylated intermediates (Figs. 1, 2) in reactions catalyzed by processive divalent cation-dependent motors. In translation, the motor is the ribosome (Trappl and Polacek 2011). In replication, the motor is DNA polymerase (Steitz 1999). In transcription, the motor is RNA polymerase (Fuchs 1976). In cellulose synthesis, the motor is glycosyl transferase (Kang et al. 1984; McNamara et al. 2015; Morgan et al. 2016). RNA and DNA retain a phosphate during polymerization, forming anionic phosphodiester linkages, while other polymers eliminate phosphate groups and form neutral linkages.

## Living Dangerously

It is a fundamental paradox of biochemistry that biopolymers are chemically unstable in their native environment—aqueous solution. All biopolymers in water spontaneously hydrolyze to the level of monomers at equilibrium in dilute aqueous solution.

### Fold

A random coil biopolymer (an ensemble of configurations) folds, by spontaneous conversion to precise three-dimensional structures characterized by specific intramolecular interactions, low configurational entropy, assignment of functional groups to exact locations and orientations in three-dimensional space, and specific interactions between functional groups. Biopolymers that occupy these precise low entropy states, stabilized by self-interactions, are called “folded.” The ability to fold is a universality of biopolymers. In our definition, a single polymer type folds. Multiple polymers of one type assemble into homogeneous assemblies. Multiple polymers of different types assemble into heterogeneous assemblies.

Elaborate folding and assembly are emergent properties of polymerization, and are possible for polymers but not for monomers. It is a universal characteristic of biopolymers that their hydrolyzed monomers do not specifically self-interact. Monomer nucleosides (Ts’o 1974), amino acids, and sugars do not pair or engage in self-complementary interactions in water. The formation of G-quadruplexes by monomeric guanosine (Gellert et al. 1962) is an exception to this universality.

**Fig. 3:**
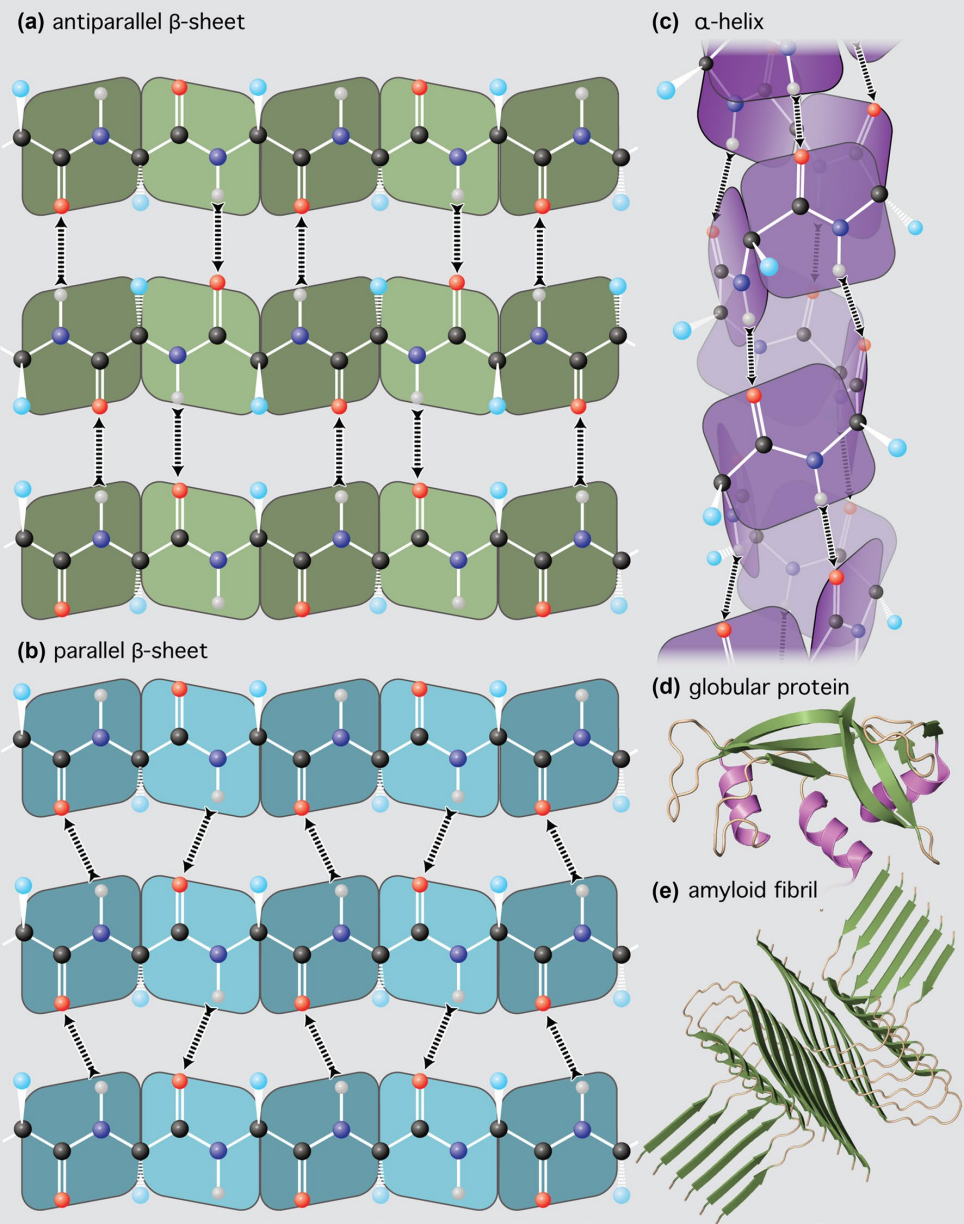
The self-complementarity of peptide linkages is the dominant molecular interaction in protein secondary structures and in folded protein. **a** Antiparallel β-sheet. **b** Parallel β-sheet. **c** α-Helix. **d** A globular protein showing α-helices (violet) and β-sheets (green). **e** An amyloid fibril showing dominance of β-sheets for any essentially amino acid sequence. Hydrogen bonding polarities are indicated by arrows. Each peptide linkage donates one hydrogen bond and accepts one hydrogen bond. (Color figure online)

Finely controlled molecular interactions allow proteins to fold into domains (Porter and Rose 2012) or fibers (Shoulders and Raines 2009) composed primarily of α-helices and β-sheets (Fig. 3) (Pauling et al. 1951; Pauling and Corey 1951; Eisenberg 2003). RNAs can fold into large domains (Woodson 2011) composed of duplexes, tetraloops, junctions, bulges, and pseudoknots (Moore 1999), which can be rigid (Ban et al. 2000) or flexible (Wan et al. 2011). Complementary DNA sequences assemble to double helices (Watson and Crick 1953) approaching a meter in length with billions of base pairs. Polyglucose assembles to microfibrils of indeterminate length containing multiple chains (Valeri 2010; Cosgrove 2014).

### Assemble

Biopolymers form heterogenous assemblies, containing multiple types of biopolymers, with specific three-dimensional structures and intermolecular interactions. For example, the prokaryotic ribosome is a heterogeneous assembly of three large rRNAs and around 50 ribosomal proteins. Nucleosomes, which are specific to eukaryotes and some archaea, are protein–DNA assemblies. Protein–saccharide assemblies are critical in cell–cell communication, cell adhesion, and host–pathogen interactions.

### Degrade

Biopolymers are ephemeral. It is a universality that biopolymers hydrolyze in aqueous media and suffer a variety of other chemical assaults in vivo and in vitro, spontaneously degrading to the level of monomers and beyond. In dilute aqueous solution, degradation of biopolymers to monomers is *always* favored in the thermodynamic sense. However, biopolymers can persist for extended periods of time in non-equilibrium states via kinetic trapping; rates of degradation are reduced by folding and assembly. These phenomena increase the depths of the kinetic traps and decrease rates of hydrolysis and other chemical degradation (McKinley et al. 1983; Prusiner 1998; Nahvi et al. 2002; Shoulders and Raines 2009; van der Lee et al. 2014). The mechanisms of decrease in rates of hydrolysis are by depopulating high energy states along degradation reaction coordinates. Folding and assembly destabilize intermediates and transition states of biopolymer hydrolysis reactions.

Therefore, it is accurate to describe biopolymers as self-protective (by folding and homogeneous assembly) and partner-protective (by heterogeneous assembly). Biopolymers are both selfish and nurturing. These properties delay but do not avert the ultimate fate of any biopolymer—hydrolysis.

Folding-based and assembly-based protection from hydrolysis, allowing persistence in aqueous environments, is pre-programmed into biopolymers at several levels. At the most fundamental level, biopolymer backbones are pre-organized for folding by geometrically arrayed, self-complementary molecular interactions and geometric propensities to fold, induced by rotameric and steric restraints on conformation. Protein conformation is restrained by the planarity of the peptide linkage and by ϕφ restraints (Pauling and Corey 1951; Ramachandran and Sasisekharan 1968). Polynucleotides are restrained by planarities of bases and by “rigidity” of nucleotides. The available conformational space of the backbone is restricted by constraints on and correlations between torsional angles (Sundaralingam and Westhof 1979). Polysaccharides are restrained by conformational preferences within and between sugars (Stick and Williams 2010). Thus, even as random coil, in which high temperature or chemical denaturants disrupt intramolecular interactions, biopolymers retain a kinetic and thermodynamic propensity to fold. Folding is fast and spontaneous when the temperature is lowered or the denaturant is removed.

## Complements to the Chef

### Complementarity

Self-complementarity is a universality of biopolymers. Self-complementarity is proficiency for preferential self-binding, which is the ability to attract and associate with self to the exclusion of non-self. Three-dimensional structures of folded/assembled DNA, RNA, protein, and polysaccharide reveal extensive networks of highly specific molecular interactions in which biopolymers complement themselves.

**Fig. 4:**
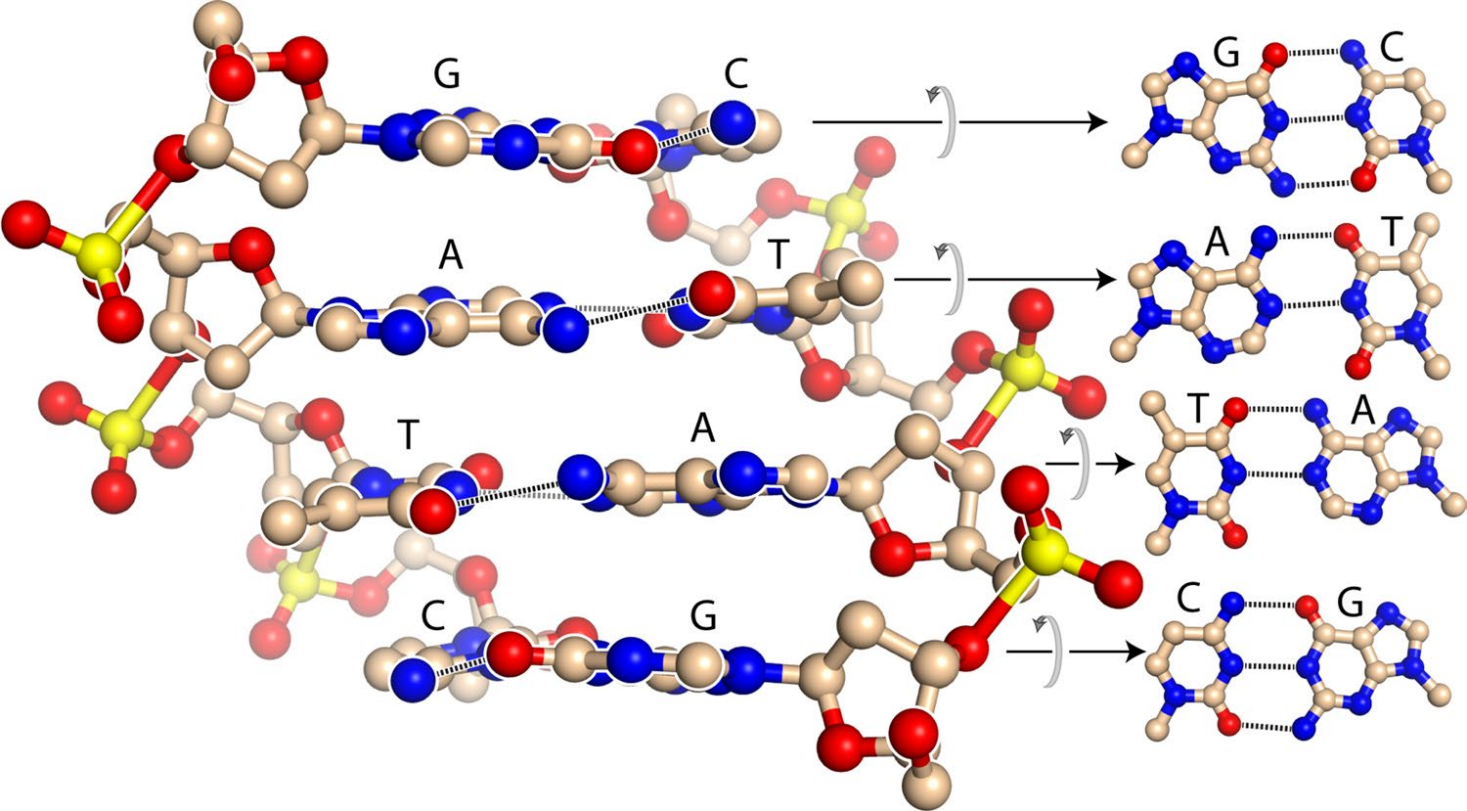
The DNA duplex with sequence GATC in each strand is self-complementary, containing geometrically matched arrays of hydrogen bond donors and acceptors that link the two strands. On the left, the normals of the base pairs are within the plane of the page. On the right the normals of the base pairs are orthogonal to the page. Hydrogen bonds are indicated by dashed lines

The term “self-complementary” has traditionally referred only to the interactions between nucleic acid bases, such as those in the DNA duplex shown in Fig. 4. “Self-complementary” has not, to our knowledge, been used previously to describe the polypeptide backbone, apparently because the nomenclature for intramolecular interactions of nucleic acids is historically distinct and separate from that describing interactions of proteins. However, “self-complementary” is an exact and accurate description of the polypeptide backbone. Polypeptide selectively adheres to itself via extended arrays of hydrogen bond donors and acceptors that are geometrically matched in three-dimensional space. This donor/acceptor matching is realized by local interactions within α-helices, or by non-local interactions within β-sheets (Fig. 3). Thus, protein realizes self-complementarity in two fully distinct folded states, a remarkable feat.

**Fig. 5:**
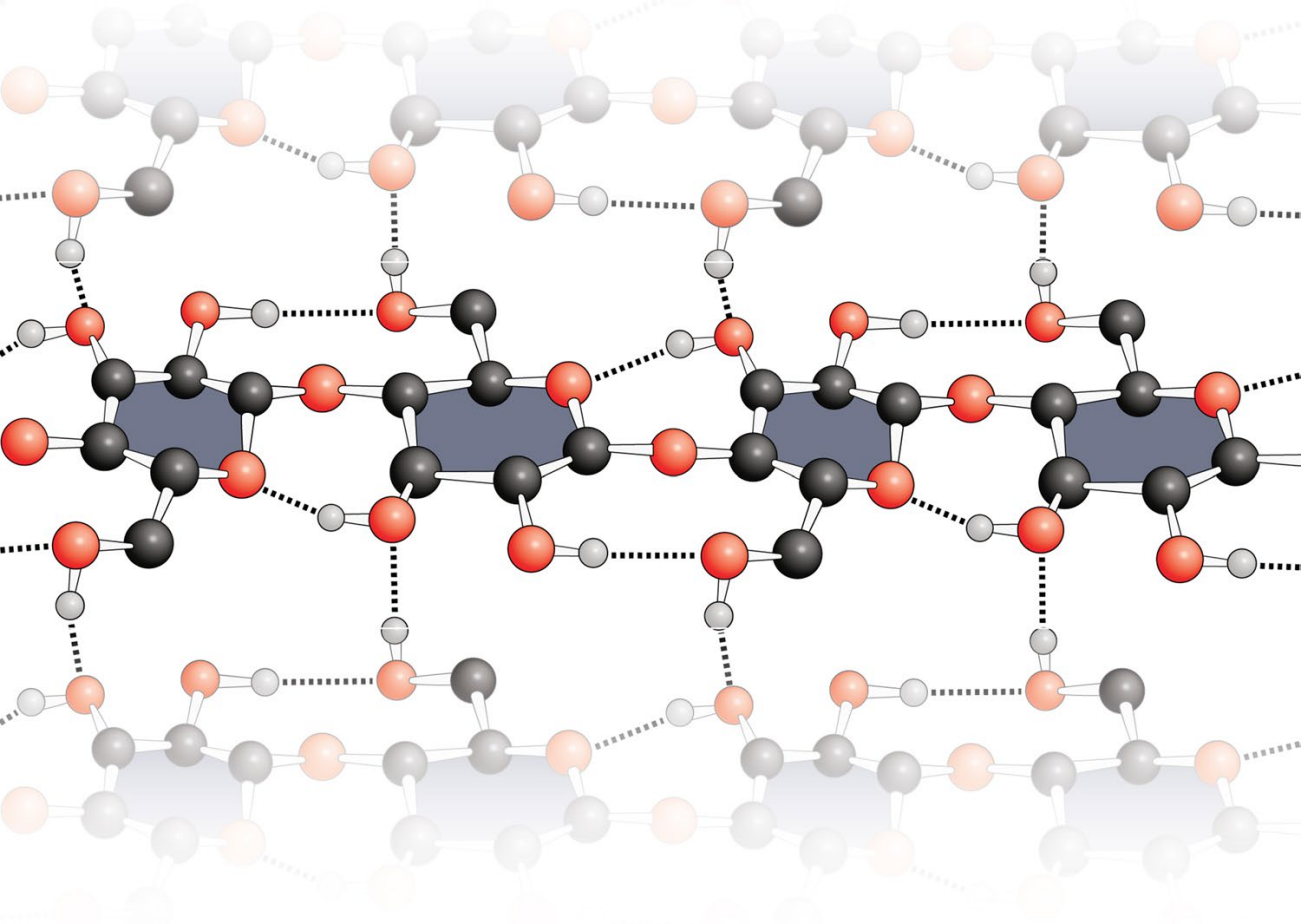
In cellulose, matched arrays of hydrogen bond donors and acceptors stabilize the folding of (1–4) polymerized glucose into homogeneous fibers. The β-anomer but not the α-anomer enables complementary glucose–glucose interactions in the polymer

Glucose in the polymerized state is intrinsically self-complementary. In cellulose, essentially all hydrogen bonding functionalities of each glucose are positively engaged with those of other glucose moieties (Fig. 5). Cellulose and chitin form stable intra-chain interfaces secured by large complementary arrays hydrogen bond donors and acceptors.

Self-complementarity of biopolymers is conditional and can be switched between “on” or “off” states. For DNA and RNA, self-complementarity is conditional on nucleotide sequence, which can act as a controlling switch for the formation of elongated DNA duplexes or RNA stem-loops. Sequences such as r(CGA–UCG) use self-complementarity to form intramolecular stem-loops or intermolecular dimers, while those such as r(CGA–CGA) do not. Complementarity is achieved by hydrogen bonding interactions between nitrogenous bases, and is switched on and off by changing the sequence. The self-complementarity of protein is modulated by amino acid composition. Specifically, proline can switch the self-complementarity to the off state by unbalancing the ratio of hydrogen bond donors to acceptors. The anomeric linkage provides the on/off switch for the self-complementarity of polyglucose. β-Anomers such as cellulose and chitin are self-complementary. α-Anomers such as glycogen and amylose are not.

Molecular interactions that enable self-complementarity of DNA, RNA, or protein primarily utilize unipolar hydrogen bonds such as those of keto oxygens, amide and imine nitrogens, and polarized amino groups (Figs. 3, 4). The molecular interactions of polysaccharides are dominated by hydroxyl groups (Fig. 5). Hydroxyl groups are bipolar, with the ability to both donate and accept hydrogen bonds.

### Perturbation—Clarification

Although certain amino acids (such proline) profoundly alter self-complementarity of polypeptide, amino acid sequence should be seen as a second-order perturbation of cohesive backbone interactions. Anfinsen described the native state of a globular protein as unique, stable, kinetically accessible, at a free energy minimum, and determined *only* by amino acid sequence (Anfinsen et al. 1961). However, essentially any amino acid sequence at high concentration forms fibrils in which β-sheet is the default mode of self-interaction (Fändrich and Dobson 2002; Pedersen et al. 2010). Globular proteins in dilute solution and amyloids at high concentrations follow the same organizing principle; both demonstrate the dominance of cohesive backbone interactions under all non-denaturing conditions.

## Separated at Birth

Although DNA, RNA, protein, and polysaccharide have many chemical and structural similarities, they are distinguished by obvious differences. The backbone of protein is neutral, cohesive, and self-complementary, enabling formation of hydrophobic cores where water is excluded. The backbones of RNA and DNA are anionic and self-repulsive. RNA folds to globular structures with wet, salty cores, while DNA tends not to form globular structures at all. Polyglucose forms dry but hydrophilic cores stabilized by the vastness of the contact area. Protein and polysaccharide folding are largely independent of cofactors. RNA and DNA folding are dependent on cationic cofactors. The specific ordering of sidechains along monotonous backbones of RNA, DNA, and proteins are important devices for modulating and manipulating conformation and molecular interaction. Protein sidechains are many and chemically diverse. RNA and DNA sidechains are few and are chemically homogeneous. Polysaccharides lack sidechains altogether. RNA, DNA, protein, and some types of polyglucose (cellulose, amylose and chitin) are linear, while glycogen (animals) and amylopectin (plants) are branched. Each of the linear biopolymers folds to helical structures (Pauling et al. 1951; Pauling and Corey 1951; Watson and Crick 1953). Polypeptide has been selected by nature to fold predominantly via backbone interactions. Polynucleotides have been selected to fold predominantly via sidechain interactions. Evolution may have found it advantageous to include additional mechanisms for modulating biopolymer properties; post polymerization modifications of biopolymers can modulate their physicochemistry and biological functions.

### Adding It Up

The net hydrogen bonding polarities of polypeptides sum to zero, with equivalent numbers of hydrogen bond donors and acceptors. Polyglucose has an excess of hydrogen bond acceptors over donors. Polynucleotides have a large excess of acceptors over donors.

### Functional Distance

Is it possible to relate the functional roles of biopolymers to their structures? First, one must attempt to accurately describe biological functions. What does each biopolymer type do? There are no bright lines—functional roles are not rigidly proscribed by polymer type. The enormous diversity in the chemical transformations of biological systems are catalyzed and regulated primarily by proteins. Protein contributes enzymes, enzyme inhibitors, structural fibers, adhesives, pumps, pores, switches, and receptors. RNA is used for temporal and specific information transfer (i.e., mRNA) and performs more limited, but nonetheless critical, catalytic functions, for example in the ribosome. By contrast, DNA appears to be used exclusively for long-term and bulk information storage (i.e., whole genome) and transfer. On the whole, polynucleotides maintain, record, read and transmit sequence information. Polysaccharides contribute structure along with energy storage and elaborate recognition.

Ribozymes (Kruger et al. 1982; Guerrier-Takada et al. 1983), which are RNA-based “enzymes,” have correctly assumed a great deal of symbolic significance and importance in discussions of fundamentals of biology and the origin of life. However, thus far there has been no observation of a biological RNA-only ribozyme that is formally enzymatic; there are no RNA-only biological ribozymes that turn over (Kruger et al. 1982; Hutchins et al. 1986; Prody et al. 1986). All RNA-only catalytic elements discovered thus far in biological systems perform suicide (single turn-over) phosphoryl transfer functions. By contrast, highly abundant and critically important ribonuclear protein ribozymes (protein-assisted ribozymes), with RNA-only catalytic sites, do turn over and are thus fully enzymatic. These RNP ribozymes include the ribosome (Khaitovich et al. 1999), RNase P (Guerrier-Takada et al. 1983), and the spliceosome (Brody and Abelson 1985). No catalytic function of polysaccharide has been observed thus far, to our knowledge.

## Fraternal Twins: DNA and RNA

DNA and RNA both fold and assemble to form double helices with central cores of paired and stacked nucleobases, framed by external, anionic backbones. DNA and RNA appear similar in chemical representations, differing only by a single atom on the backbone and by a methyl group on one base.

**Fig. 6:**
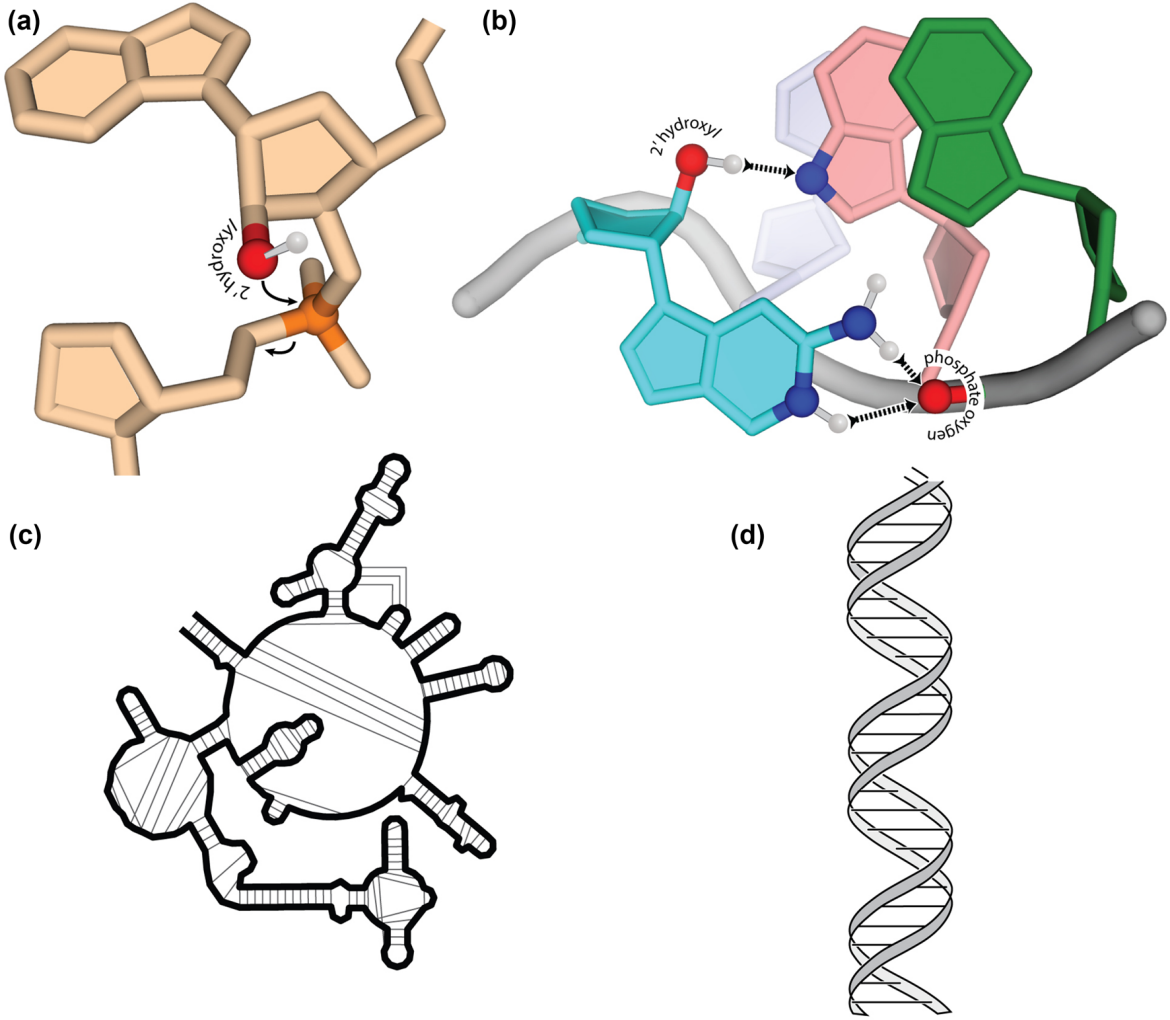
The impact of the 2′ hydroxyl group on polynucleotide reactivity and structure. **a** Reactivity. RNA holds a gun to its own head. The 2′ oxygen is a nucleophile that is poised to attack the adjacent phosphorous atom, cleaving the RNA backbone. **b** Folding. The 2′ hydroxyl group nucleates folding of complex structures by enabling hydrogen bonding between the backbone and bases, as demonstrated in the GNRA tetraloop. The 2′ hydroxyl of a guanine forms a hydrogen bond with the N7 of an adenine. In addition, the N1 and N2 of the guanine form a hydrogen bond with a phosphate oxygen of the backbone. **c** Complexity. RNA folds into elaborate three-dimensional structures. **d** DNA folds to long double helices. In panels a and b, hydrogen bonding groups that do not form hydrogen bonds are omitted for clarity

The 2′ hydroxyl group profoundly influences folding, providing a nucleation hook for base–backbone association, thus fostering diverse loops and junctions. The preponderance of hydrogen bond acceptors over donors of DNA is partially relaxed in RNA by the 2′ hydroxyl group, which provides a locus for intramolecular cohesion. A frequent folding motif involving base–backbone interactions of rRNA is the GNRA tetraloop. There are over 40 examples of this motif in the large ribosomal subunit of prokaryotes (Hsiao et al. 2009). These structures, and many other non-helical structures, are stabilized by intramolecular interactions between 2′ hydroxyl groups and RNA bases (Fig. 6b). These base–backbone interactions promote folding of RNA into local stem-loops, which are often further stabilized by tertiary interactions (Fig. 6c). Biological DNA, by contrast, is generally restrained to base–base associations, forming long, monotonous double helices (Fig. 6d).

Profound differences in reactivity distinguish RNA from DNA. RNA is recalcitrant to oxidative radical damage relative to DNA. However, RNA “holds a gun to its own head”; each 2′ hydroxyl of RNA is poised for nucleophilic attack at the adjacent phosphorus atom, causing cleavage of the backbone (Fig. 6a). The rate of RNA self-cleavage is modulated by local structure, flexibility, pH, and interactions with cations. Thus, the RNA and DNA backbones have distinctive lability profiles, which depend on many factors including on the chemistry of the cleavage process.

## Nature Chose Phosphate

Westheimer suggested that phosphates dominate molecular biology because phosphate is a kinetically trapped (e.g., a phosphate ester), tunable, water-soluble leaving group that can be linked to small molecules, conferring anionic charge and blocking transit across membranes (Westheimer 1987). While correct, in our view this analysis should be extended to incorporate the role of phosphate in mechanochemical coupling.

All biopolymerization reactions utilize phosphorylated or pyrophosphorylated intermediates (Figs. 1, 2) in reactions catalyzed by processive enzymes. Phosphorylated intermediates appear to be necessary for the mechanochemical coupling required for processive polymerization. The polymerases that make DNA, RNA, protein, and polysaccharide are nanoscale motors. Translocation is energy-driven; the nascent polymer translocates relative to the polymerization enzyme. Mechanochemical coupling in motor proteins is commonly linked to association/dissociation of phosphate because phosphate has “claws” that reach out in three dimensions; phosphate can grab onto and deform proteins. The strength, directionality, and unipolarity of hydrogen bonding and electrostatic interactions between phosphate and protein cause linkage of phosphate association to protein conformation (Rice et al. 1999; Wittinghofer 2016). This coupling of directed molecular displacement (work) to association/dissociation of phosphate, which is in turn linked to pyrophosphate hydrolysis, has been characterized in myosin and kinesin, in the ribosome and in DNA, RNA, and cellulose polymerases (Wang et al. 1998; Morin et al. 2015; Morgan et al. 2016; Arias-Gonzalez 2017). During polymerizations of DNA, RNA, protein, and polyglucose, translocations are structurally and energetically coupled to phosphate association/dissociation.

## Molecules in Mutualism

We (Williams) have previously proposed that formalisms for describing mutualisms on levels of cells, organisms, and ecosystems also apply to biopolymers (Lanier et al. 2017). Mutualisms are everywhere in the biosphere and are fundamentally important in evolution, ecology, and economy (Moran 2006; Bronstein 2015; Douglas 2015; Gray 2017). The mutual benefit, exchange of proficiencies, persistence, interdependence, co-evolution, and parasitism that characterize relationships on cellular, organismal, and ecological levels have direct parallels in the behaviors of biopolymers.

A mutualism is a persistent and intimate interaction that benefits multiple interactors (Douglas 2015). Because mutualisms are prolonged and intimate, partners in mutualism influence each other’s evolution. Evolutionary change of one partner triggers change of the other. We believe that biopolymers are mutually imprinted on each other in structure and function via their co-evolution, stabilizing the mutualism.

### Levels of Mutualism

Mutualisms were previously understood to operate at the levels of cells, organisms, ecosystems, and even societies and economies. The eukaryotic cell is a culmination of mutualism between simpler prokaryotic cells (Sagan 1967; Poole and Gribaldo 2014; Gray 2017). Essentially every species on Earth is involved in mutualisms.

### Molecules

Biopolymers satisfy all of the formalisms of mutualism. Biopolymers protect each other from hydrolysis and synthesize each other. Polypeptide synthesizes polynucleotide (polymerases) and polynucleotide synthesizes protein (the ribosome). During essential steps of translation, coding is performed by proteins (aaRS enzymes charge tRNAs), while decoding is performed by RNAs (mRNA and rRNA) in the ribosome. Molecules in Mutualism describes: (i) survival—extant biopolymers are more persistent than competing polymer types, which are now extinct; (ii) co-evolution—biopolymers created each other in an emergent and cooperative environment of chemical evolution; (iii) fitness—biopolymers are more ‘fit’ in combination than in isolation; (iv) distance—each biopolymer type has distinct proficiencies and chemical characteristics; (v) innovation—proficiencies of one type of biopolymer release constraints on partner biopolymer types; (vi) robustness—biopolymer types have been fixed for billions of years, meaning biopolymers compose seminal and ancient mutualism with profound stability; and (vii) parasitism—examples of molecular self-interest and escape from mutualism are seen in amyloids (McKinley et al. 1983) and phase-separated RNA gels (Jain and Vale 2017).

## Origins of Biopolymers—Origins of Life

### Why Biopolymers?

Biology requires polymers. Biopolymers allow processes of folding and assembly to be detached from the required investment of free energy. For biopolymers, prior free energy investment in synthesis is distributed over time and space, offsetting the subsequent cost of folding and assembly. Biopolymers appear to *spontaneously* fold and assemble, only because of prior free energy investments. For small molecules, by contrast, assembly and investment are directly coupled. The free energy of assembly is paid in real time, during molecular assembly. Therefore, small molecules cannot achieve the elaborate folds and assemblies, based on conditional self-complementarity, that appear to come naturally to biopolymers.

The data surveyed here suggest that polypeptide, polynucleotide, and polysaccharide arose by co-evolution. Biopolymer universalities, including (i) synthesis by condensation and degradation by hydrolysis, (ii) folding by pre-organization and self-complementarity, (iii) homogeneous and heterogeneous assembly, and (iv) protection by folding or homogeneous assembly (selfishness) and (v) protection by heterogeneous assembly (mutualism), point to simultaneous origins in a shared environment. The co-origins of biopolymers are consistent with previous reports of common chemistry of monomer formation (Miller and Urey 1959; Oró and Guidry 1960; Patel et al. 2015).

### Origins of Biopolymers

In our view, observed biopolymer universalities and idiosyncracies support a model in which polymer synthesis by condensation cooperated with hydrolytic degradation, mediated by folding and assembly, to drive chemical evolution (Brack 1987; Abkevich et al. 1996; Hud and Anet 2000; Peters and Williams 2012). In contrast to the consensus, this model suggests that early selection operated at the level of hydrolytic degradation (mitigated by folding and assembly), rather than at the level of synthesis. After nearly 4 billion years of evolution, biopolymers continue to utilize self-complementarity to escape hydrolysis and increase persistence (Prusiner 1998; Jain and Vale 2017; Bai et al. 2018).

We (Hud) have proposed that the thermodynamic driver for synthesis and degradation on the ancient Earth would have been cycling water activity (Forsythe et al. 2015), which was and is ubiquitous over the landmass of the Earth. Thus, it seems possible that polymers originated via simple (non-redox) chemistry that remained near equilibrium, rocking gently in the cradle of day/night cycling (Hud and Anet 2000). Synthesis by condensation dehydration is favored in low water activity (day) and degradation by hydrolysis is favored in high water activity (night). The close analogy of biopolymer synthesis/degradation by hydrolysis/condensation in biochemical system to wet/dry cycling in geochemistry suggests that the origin of life, like extant life, was planet-wide phenomena of surfaces, and was not a function of exotic environments with constant, high water activity as in hydrothermal vents (Corliss et al. 1981).

### Selfish Molecules

One can define biopolymer “self-interest”. Molecular self-interest is chemical persistence. Persistence of biopolymers in a hydrolytic environment is enhanced by folding and assembly. Self-complementarity is therefore an expression of self-interest, a method to escape from hydrolysis, a path to survival, and a property universal to biopolymers. Heterogeneous assemblies are expressions of partner-protection from hydrolysis, described here and elsewhere as molecular mutualisms (Lanier et al. 2017).

The observed nominal stability, rather than extreme stability, of biopolymer folds and assemblies, suggests that unfolding and disassembly confers advantage in some circumstances. The ability to unfold and disassemble provides pathways for prospecting for new folds and new partners and for recycling. Extremely stable folds and assemblies could persist for some period but ultimately form molecular dead-ends.

### Losers

The juxtaposition of biopolymer universalities next to the diversity of chemical compositions of multiple biopolymer types is consistent with a model of simultaneous biopolymer origins via step-wise evolutionary processes, rather than from direct but improbable and singular phenomena (Ricardo et al. 2004). It seems likely that our small set of surviving biopolymers were chemically selected from diverse competing polymers (Hud et al. 2013), most of which failed to compete successfully because of their lesser ability to fold and assemble. Biopolymers, as indicated by spider webs, DNA nanodevices, chromatin, the ribosome, and cellulose, are masters of folding and assembly. It seems improbable that this mastery arose from good luck, rather than from chemical evolution. Loser polymer types, which were less accomplished at folding and assembly, were forced into hydrolytic extinction.

If so, ancestral polymers, which dominated in early stages, would have been supplanted by more successful second- or third-generation polymers. The scenario described here does not ascribe utility to catalysis or replication during the early origins of biopolymers and is agnostic on compartmentalization (Szostak 2017), although it does seem to require that compartments be competent to tolerate cycling water activity.

Biopolymer universalities are not inconsistent with conclusions of de la Escosura and coworkers, who argue that the origin of life involved a “system” (de la Escosura et al. 2015). Their *system*, a heterogeneous, functionally integrated, self-maintained, quasi-stationary state allowing for increases in complexity and elaboration, is a chemically vague but reasonable description of our shared environment of cycling water activity and co-evolution, with chemical selection at the level of degradation.

## Conclusion

Although biopolymer types are traditionally studied and taught in isolation of each other, we believe that DNA, RNA, polypeptide, and polysaccharide are best understood in the context of their shared attributes and key differences. Recognition of biopolymer universalities explains their structures and functions and points to their origins. Foundational among these universalities is the ability of all biopolymers to fold via self-complementarity and assemble into structures that protect them (at least for a while) from their thermodynamic fate of chemical degradation in dilute aqueous solution. Only by examining biopolymers in context can we hope to achieve a reasonable understanding of the fundamental molecules of life.
